# Data on effects of rotenone on calcium retention capacity, respiration and activities of respiratory chain complexes I and II in isolated rat brain mitochondria

**DOI:** 10.1016/j.dib.2017.06.052

**Published:** 2017-07-04

**Authors:** Evelina Rekuviene, Laima Ivanoviene, Vilmante Borutaite, Ramune Morkuniene

**Affiliations:** a Neuroscience Institute, Lithuanian University of Health Sciences, Eiveniu 4, LT-50161 Kaunas, Lithuania; b Department of Biochemistry, Lithuanian University of Health Sciences, Eiveniu 4, LT-50161 Kaunas, Lithuania

**Keywords:** Rotenone, Brain ischemia, Mitochondria, Calcium retention capacity, Complex I, Respiration

## Abstract

The data presented in this article are related to the research article entitled “Rotenone decreases ischemia-induced injury by inhibiting mitochondrial permeability transition in mature brains” (Rekuviene et al., 2017) [1]. Data in this article present the direct effects of rotenone on calcium retention capacity (CRC) in isolated normal cortex and cerebellum mitochondria, effects of rotenone intravenous infusion on leak and phosphorylating respiration rates of isolated cortex and cerebellum mitochondria, on activities of respiratory chain complexes I and II in freezed-thawed/sonicated cortex and cerebellum mitochondria after brain ischemia. In addition, detailed experimental procedures of isolation of brain mitochondria, measurements of CRC, respiration, activities of respiratory chain complexes and H_2_O_2_ generation in cortex and cerebellum mitochondria are described.

## Specifications Table

**Table:** 

Subject area	*Biology*
More specific subject area	*Biology of brain disorders*
Type of data	*Figures, Table*
How data was acquired	*CRC was determined fluorimetrically (Fluorescence Spectrometer Perkin Elmer LS55).*
	*Mitochondrial respiration was measured with high-resolution respirometry OROBOROS Oxygraph-2 k (Oroboros Instruments, Innsbruck, Austria).*
	*Respiratory chain complex I and complex II activities were determined spectrophotometrically (Nanophotometer).*
Data format	*Analyzed*
Experimental factors	*CRC of isolated normal brain mitochondria was measured with directly added rotenone (50–1000 nM), brain mitochondrial respiration and activities of respiratory chain complexes I and II were measured after infusion of single rotenone dose (0.01 mg/kg) into the tail vein of rats and the exposure of 120 min brain ischemia.*
Experimental features	*Fluorimetric determination of mitochondrial CRC with Calcium Green-5N, spectrophotometric determination of activities of respiratory chain complexes I (by NADH oxidation) and II (by 2,6-dichlorophenolindophenol reduction) in freezed-thawed/sonicated mitochondria, respirometric measurements (oxygen consumption rate) of isolated brain mitochondria.*
Data source location	*Kaunas, Lithuania*
Data accessibility	*The data are available with this article*

## Value of the data

● Data from this research highlights the effects of rotenone on rat brain mitochondrial functions.
● The direct effects of rotenone (50–1000 nM) on CRC of isolated normal brain cortex and cerebellar mitochondria were measured.
● The effects of the intravenous infusion of rotenone (0.01 mg/kg) on leak and phosphorylating respiration of isolated cortex and cerebellum mitochondria as well as on activities of mitochondrial respiratory chain complexes I and II after 120 min brain ischemia were measured.
● These data may be relevant for (i) other researchers using various doses of rotenone in their experiments with mitochondria; (ii) research that focuses on the mitochondrial respiratory chain complex I inhibition during brain ischemia.

## 1 Data

**Fig. 1:**
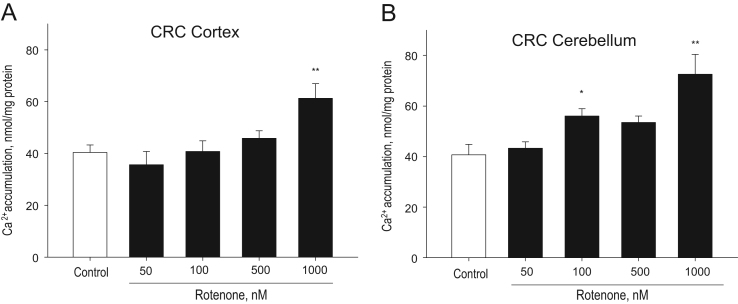
Direct effect of rotenone on CRC of isolated cortex (A) and cerebellum (B) mitochondria ** – *p*<0.01, * – *p*<0.05, if compared to control. Means±standard errors of 4–7 separate experiments are presented.

**Fig. 2:**
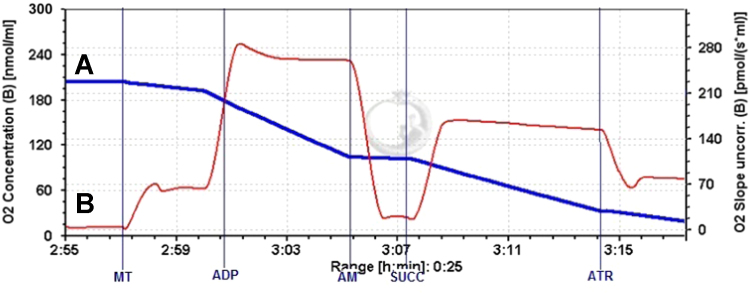
Typical traces of respirometric recording of cortex mitochondria Additions: MT – mitochondria (0.25 mg protein/mL) in the presence of pyruvate/malate (1/1 mM); ADP – 2 mM; AM – 1.5 mM amytal; SUCC – 5 mM succinate; ATR – 100 μM atractyloside. A trace indicates oxygen concentration (nmol/ml), B – oxygen flux (pmol O_2_/(s˟ml).

**Fig. 3:**
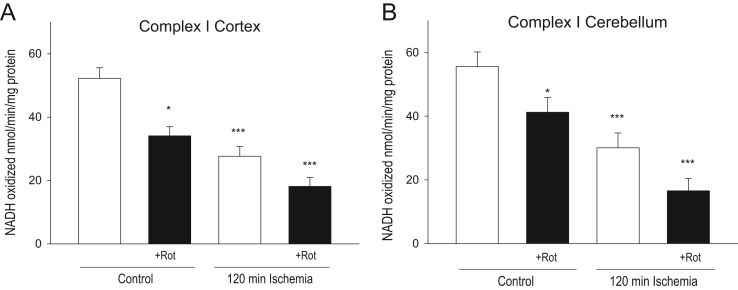
Effect of rotenone intravenous infusion on complex I activity of cortex (A) and cerebellum (B) control and ischemia damaged mitochondria * – *p*<0.05, *** – *p*<0.01, if compared to control. Means±standard errors of 3–8 separate experiments are presented.

**Fig. 4:**
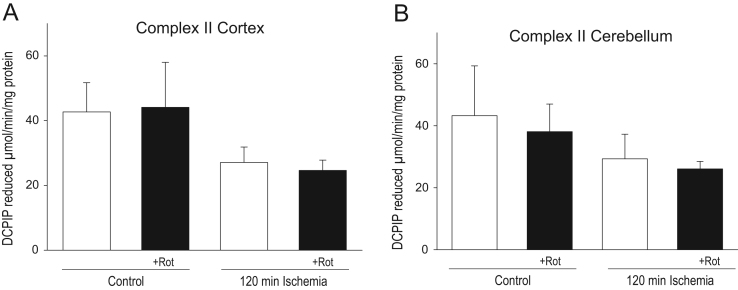
Effect of rotenone intravenous infusion on complex II activity of cortex (A) and cerebellum (B) control and ischemia damaged mitochondria Means±standard errors of 3 separate experiments are presented.

The data reported include direct effects of rotenone (50–1000 nM) on calcium retention capacity (CRC) in isolated control cortex and cerebellum mitochondria (Fig. 1). We also measured the effects of intravenous rotenone infusion (0.01 mg/kg) on respiration rates (leak and phosphorylating) of isolated normal and 120 min ischemia damaged cortex and cerebellum mitochondria respiring with substrates pyruvate/malate and succinate (Table 1) as well as original recording of respirometric curve of mitochondria is presented (Fig. 2). Effects of rotenone intravenous infusion (0.01 mg/kg) on complex I (Fig. 3) and complex II (Fig. 4) activities of mitochondria isolated from control rat cortex and cerebellum and after 120 min brain ischemia are shown. The detailed experimental procedures of isolation of brain cortex and cerebellum mitochondria, measurements of CRC, respiration, activities of respiratory chain complexes I and II and H_2_O_2_ generation in cortex and cerebellum mitochondria are described.

**Table 1:** Effect of rotenone intravenous infusion on respiration of control and ischemia damaged cortex and cerebellum mitochondria. Rates of oxygen consumption are expressed in pmol O/s/mg mitochondrial protein. *V*_Leak_ – mitochondrial leak respiration in the presence of pyruvate/malate (1/1 mM) and mitochondria (0.25 mg/ml); *V*_ADP_ – mitochondrial phosphorylating respiration with ADP (2 mM) and indicated substrate (either pyruvate/malate or succinate/amytal); *V*_Atr_ – atractyloside inhibited respiration in the presence of succinate (5 mM) and amytal (1.5 mM). *** – *p*<0.001, ** – *p*<0.01, * – *p*<0.05, if compared to control; # – *p*<0.05, if compared to 120 min ischemia group. Means±standard errors of 3–7 separate experiments are presented.

**Cortex**				
*Pyruvate/malate*	Control	Rot Control	Ischemia	Rot+Ischemia
V_Leak_	199.1±14.2	154.05±9.33*	78.6±4.7***	121.45±20.25#
V_ADP_	851.0±45.1	573.66±84.58**	132.9±19.0***	181.54±31.18#
*Succinate*				
V_ADP_	530.7±29.3	525.29±29.56	107.0±8.0***	173.49±31.25#
V_Atr_	301.1±9.4	330.08±30.43	126.5±18.7***	163.98±35.31#

**Cerebellum**				
*Pyruvate/malate*	Control	Rot Control	Ischemia	Rot+Ischemia

V_Leak_	164.9±9.8	131.35±5.43*	113.6±8.3**	128.39±30.25
V_ADP_	857.1±40.1	568.16±64.89**	222.8±25.6***	231.76±66.41
*Succinate*				
V_ADP_	534.8±13.5	422.92±51.02	164.8±11.5*	200.11±57.51
V_Atr_	318.5±9.5	272.04±28.99	156.1±11.5*	194.79±58.40

⁎⁎⁎ - p<0.001,

⁎⁎ - p<0.01,

⁎ - p<0.05, if compared to control;

# - p<0.05, if compared to 120 min ischemia group. Means ± standard errors of 3–7 separate experiments are presented.

## 2 Experimental design, materials and methods

### 2.1 Materials

Calcium Green-5N and Amplex Red were obtained from Molecular Probes/Invitrogen (USA), HBSS was from GIBCO (UK). All other materials were purchased from Sigma-Aldrich (Germany).

### 2.2 Isolation procedure of mitochondria from cortex and cerebellum

All procedures of mitochondrial isolation were performed on ice. Rat brains, cortex and cerebellum, were placed in isolation medium (containing 225 mM mannitol, 75 mM sucrose, 5 mM HEPES, 1 mM EGTA, pH 7.4) and homogenized using a glass-teflon homogenizer. The tissue homogenate was spun 5 min×1000*g* and 10 min×10,000*g* with centrifuge Heraeus Biofuge Stratos (Thermo Fisher Scientific). After centrifugation, mitochondrial pellet was suspended in isolation medium and total mitochondrial protein was determined by the modified Biuret method [2].

### 2.3 Measurement of calcium retention capacity in isolated mitochondria

CRC of isolated brain mitochondria was determined fluorimetrically (Fluorescence Spectrometer Perkin Elmer LS55) using fluorescent dye Calcium Green 5N 100 nM (excitation at 507 nm, emission at 536 nm). Mitochondria (0.2 mg/ml) were incubated in a buffer (200 mM sucrose, 10 mM Tris–HCl, 1 mM KH_2_PO_4_, 10 μM EGTA and 5 mM succinate, pH 7.4) at 25 °C, and repeatedly supplemented with 1.67 µM CaCl_2_ every 120 s until spontaneous release of Ca^2+^ caused an increase in fluorescence indicating mitochondrial permeability transition pore (mPTP) opening referred as CRC. In experiments on direct effects of rotenone, isolated control mitochondria were incubated for 2 min in the presence of various concentrations of rotenone (50–1000 nM) then pulses of Ca^2+^ were repeatedly added as described above. Calibration of the signal was achieved by the addition of known amounts of CaCl_2_.

### 2.4 Measurement of mitochondrial respiration

Mitochondrial respiration was measured with high-resolution respirometry OROBOROS Oxygraph-2 k (Oroboros Instruments, Innsbruck, Austria) at 37 °C in 2 ml respiration buffer containing 110 mM KCl, 10 mM Tris–HCl, 5 mM KH_2_PO_4_, 2.24 mM MgCl_2_, pH 7.2 and 0.25 mg/ml mitochondrial protein. The Oroboros Oxygraph-2 k system allowed simultaneous measurement of two different mitochondrial samples and two respiration substrates representing different metabolic pathways, pyruvate/malate as respiratory complex I substrates and succinate plus amytal as respiratory complex II substrate. Respiration assay started with addition of pyruvate/malate (1/1 mM) and mitochondria (0.25 mg/ml) (see original recording of respirometric curve in Fig. 2), this respiration was denominated as leak respiration (*V*_Leak_) and represented proton leak-driven respiration [3]. Then the respiration buffer was supplemented with 2 mM ADP and ADP-stimulated phosphorylating respiration (*V*_ADP_) was registered with pyruvate/malate. The sequential additions of 1.5 mM amytal and 5 mM succinate allowed to measure complex II-dependent *V*_ADP_. Finally, 100 μM atractyloside, a blocker of ADP transport into the matrix, was used and represented atractyloside- inhibited respiration (*V*_Atr_) as succinate -dependent leak respiration.

### 2.5 Measurement of activity of complex I of the mitochondrial respiratory chain

Complex I activity was determined spectrophotometrically (Nanophotometer) by following the kinetics of 100 µM NADH oxidation for 4 min at a wavelength of 340 nm in a medium containing 25 mM KH_2_PO_4_, 60 µM coenzyme Q_1_, 2 µg/ml antimycin, 2 mM sodium azide, 3 mg/ml bovine serum albumin (BSA) and 0.125 mg/ml freezed-thawed/sonicated mitochondria (pH 7.4 at 37 °C). Complex I activity was calculated as the difference between NADH oxidation rate without and with complex I inhibitor rotenone (10 μM) and expressed as nmol/min mg protein.

### 2.6 Measurement of activity of complex II of the mitochondrial respiratory chain

Complex II activity was assessed by following the reduction of 2,6-dichlorophenolindophenol (DCPIP) spectrophotometrically (Nanophotometer) by the decrease of absorbance at 600 nm of the oxidized DCPIP in buffer containing 50 mM KH_2_PO_4_, 0.1 mM EDTA, 2 mM sodium azide, 10 mM succinate, 0.125 mg/ml freezed-thawed/sonicated mitochondria, pH 7.4 and at 25 °C. The reaction was started with 1.63 mM phenazine methosulfate and 35 µM DCPIP. Complex II activity was expressed as DCPIP reduction rate, µmol/min mg protein. The extinction coefficient for DCPIP is *ε*=19.2 mM/cm.

### 2.7 Measurement of mitochondrial H_2_O_2_ generation

The measurement protocol of mitochondrial H_2_O_2_ generation presented in this article is related to the research article entitled “Rotenone decreases ischemia-induced injury by inhibiting mitochondrial permeability transition in mature brains” [1]. H_2_O_2_ generation in isolated mitochondria was estimated fluorimetrically with a Thermo Scientific Plate reader Fluoroskan Ascent in 200 µl of respiration buffer (110 mM KCl, 10 mM Tris–HCl, 5 mM KH_2_PO_4_, 2.24 mM MgCl_2_, pH 7.2) at 25 °C, with 10 μM Amplex Red and 5 U/mL horseradish peroxidase Type IV-A (excitation at 544 nm, emission at 590 nm). Mitochondria (0.05 mg/mL) were added to respiration buffer supplemented with pyruvate/malate (1/1 mM) or succinate (5 mM), or both, recording the change of fluorescence signal for 30 min. In some measurements, when succinate alone was used as a substrate, exogenous rotenone (1 µM) was added to respiration buffer. Fluorescence signal was calibrated using known amounts of H_2_O_2_. The rate of H_2_O_2_ emission was expressed in pmol/min/mg protein, which was equated as 100% of control mitochondria.

### 2.8 Statistical analysis

Sigma Plot 13 Statistics software was used for statistical analysis. Results are expressed as mean±standard errors. Statistical analysis was performed using One Way ANOVA followed by Tukey or Fisher LSD post hoc tests. A value of *p*<0.05 was considered as statistically significant result.
