# Supplementary material for: Data on effects of rotenone on calcium retention capacity, respiration and activities of respiratory chain complexes I and II in isolated rat brain mitochondria
Source: Data Brief. 2017 Jul 4;13:707–12. doi: 10.1016/j.dib.2017.06.052 (PMC5512184; doi:10.1016/j.dib.2017.06.052)
Supplement: Supplementary file 1 — Supplementary material [file mmc1.doc]

**Conflict of Interest Form**

23 June, 2017

Dear Editor,

The authors declare no competing interests.

Sincerely,

Dr. Ramune Morkuniene

Corresponding author
